# Associations between ethylene oxide exposure and chronic bronchitis: results from the NHANES 2013–2018

**DOI:** 10.3389/fpubh.2024.1424555

**Published:** 2024-12-09

**Authors:** Yan Li, Hui Wang, Xiaoqing Bi, Guowei Zhao

**Affiliations:** Department of Dermatology, ZiBo Central Hospital, Zibo, Shangdong, China

**Keywords:** ethylene oxide, chronic bronchitis, NHANES, cross-sectional study, epidemiology

## Abstract

**Introduction:**

Ethylene oxide (EtO) is a reactive gas commonly used in the production of various chemical compounds. Research has linked EtO exposure to respiratory conditions, including chronic obstructive pulmonary disease (COPD) and asthma. However, its potential effects on chronic bronchitis (CB) remain unclear, highlighting the need for further study to understand its role in respiratory health.

**Methods:**

Our study investigated data from 5,044 NHANES participants between 2013 and 2018 across three 2-year survey cycles. The relationship between HbEtO and CB was examined using weighted logistic regression, with HbEtO quartiles analyzed to assess the trend. A smoothed curve was fitted to verify the relationship between HbEtO and CB. Additionally, sensitivity analyses were conducted to assess the robustness of our results, while subgroup analyses explored potential effect modifiers of the HbEtO-CB association.

**Results:**

Compared with patients without CB, patients with CB had elevated HbEtO levels. Specifically, natural Log_2_HbEtO levels were linked to a greater prevalence of CB in a fully adjusted model (OR = 1.243, 95% CI: 1.143–1.352). Analysis of Log_2_HbEtO quartiles showed a significant trend in Q4 compared with Q1 (*p* for trend < 0.001). The fitted smoothed curve indicated a U-shaped nonlinear association, with saturation and threshold analysis revealing an inflection point at Log_2_HbEtO = 4.87. Sensitivity analyses confirmed the robustness of these findings, and subgroup analyses identified consistent associations across various groups.

**Conclusion:**

Our study found a significant association between EtO exposure and the occurrence of CB. Given the health risks linked to EtO exposure, implementing effective control measures is essential. Such actions could help lower CB prevalence and enhance respiratory health in vulnerable populations.

## 1 Introduction

Ethylene oxide (EtO) is a reactive epoxide that is an important raw material for the production of ethoxylates and other compounds used in a wide variety of industrial processes (1). It is present in everyday products such as disinfectants for medical equipment, fumigants and chemicals used in the production of cosmetics, detergents and pharmaceuticals (2). Ethylene oxide is one of the most produced organic compounds, with a global production of more than 20 million tons. Due to its wide industrial application, people are exposed to ethylene oxide not only in occupational settings (3, 4), but also through environmental contamination (e.g., air pollution and contaminated food or water). In the human body, ethylene oxide is rapidly absorbed by inhalation and distributed throughout the body, forming adducts with hemoglobin (5, 6) and DNA (7, 8). Although adverse health effects of ethylene oxide have been extensively documented in animal models (2, 9, 10), the effects on human health, particularly on non-cancer outcomes such as chronic bronchitis (CB), remain under-explored. Prolonged exposure to ethylene oxide for industrial workers or those living near ethylene oxide-emitting facilities may lead to long-term health consequences (11–14).

CB is a common respiratory disease affecting 12 to 16 million individuals in the United States (15), where it has consistently been the third leading cause of death since 2008 (16, 17). CB is marked by persistent coughing and mucus production that typically last about 2 years, with symptoms recurring for at least 3 months each year. This condition can accelerate lung function decline, increase rates of exacerbation, and potentially raise overall mortality. CB is an important public health problem affecting millions of people worldwide. CB is characterized by persistent cough and sputum, usually due to prolonged exposure to irritants. Although smoking is the main risk factor, environmental pollutants such as ethylene oxide may also contribute to the pathogenesis of CB (18, 19). However, further direct studies are needed to more fully understand the mechanisms by which ethylene oxide affects human health.

The median prevalence of CB in Europe is 2.6%. While smoking accounts for only 30% of this prevalence, other factors, such as EtO exposure, may also play a significant role (20). In 2016, the U.S. Environmental Protection Agency (USEPA) re-evaluated the toxicity of EtO, more than doubling the unit risk level from previous estimates. This has raised public awareness and concerns over the health risks associated with EtO exposure. Prolonged exposure to EtO is linked to cancer and neurological disorders, and it can also lead to symptoms like nausea, pulmonary edema, and bronchitis (21, 22). Despite these recognized effects, the relationship between EtO exposure and bronchitis remains underexplored. Given the respiratory risks associated with frequent EtO exposure, understanding its health impact is essential. This study, therefore, utilized data from a U.S. population sample to examine the potential link between EtO exposure and CB.

## 2 Methods

### 2.1 Study population

This study utilized data from the National Health and Nutrition Examination Survey (NHANES), a program designed to assess various aspects of health and nutrition in the U.S. population. To accurately reflect the population's overall health, NHANES employed a stratified, multistage probability sampling method (23). Data for this analysis were sourced from the NHANES public database, available at https://wwwn.cdc.gov/nchs/nhanes (24).

**Figure 1:**
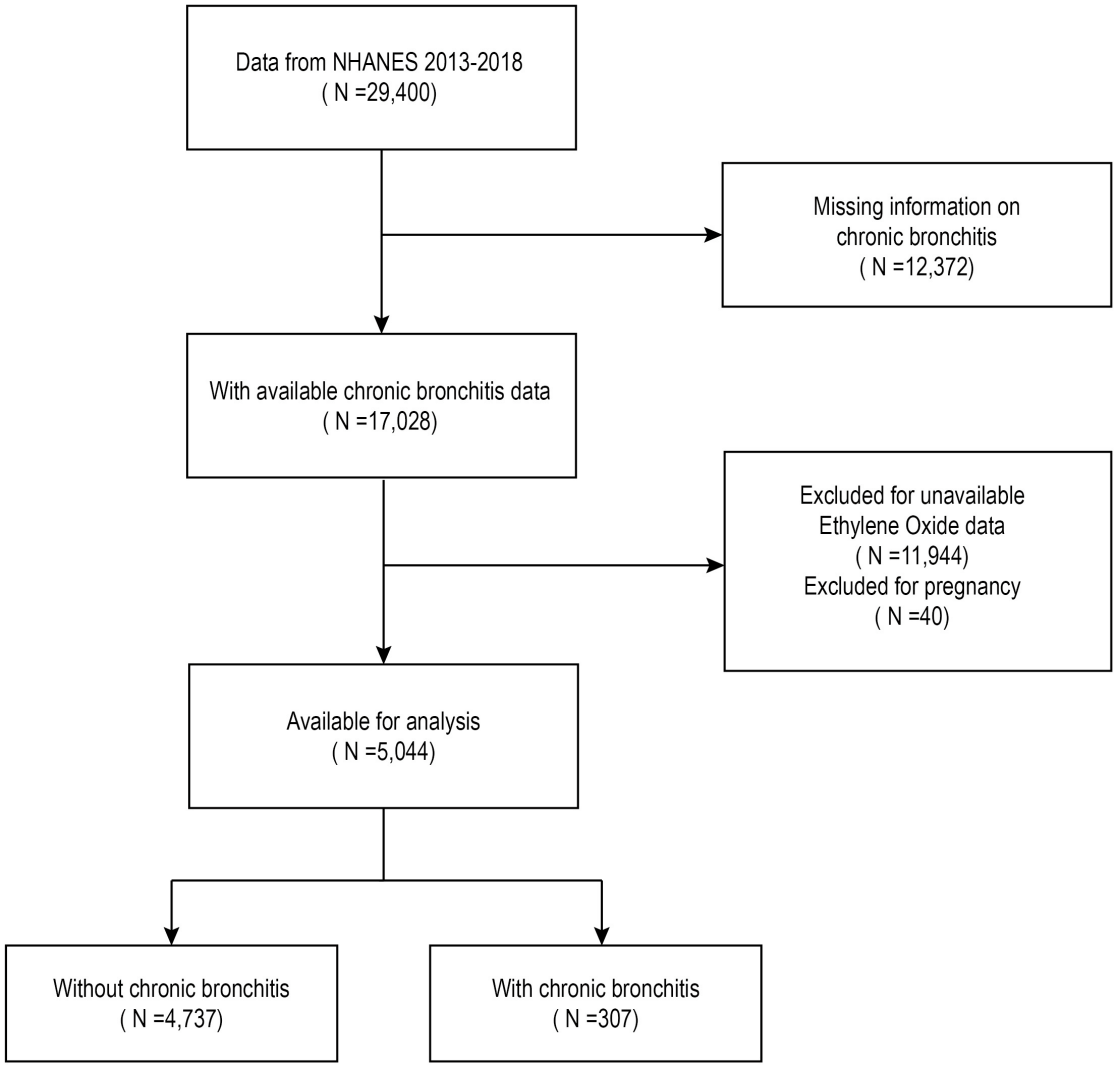
The participant selection flowchart is based on the NHANES database.

The analysis included data from 29,400 individuals surveyed between 2013 and 2018. Of these, 40 individuals who reported being pregnant, 12,372 with no data on CB, and 11,944 without hemoglobin adduct of ethylene oxide (HbEtO) data were excluded. After these exclusions, the final sample consisted of 5,044 individuals, including 307 with CB and 4,737 without (Figure 1).

### 2.2 The definition of CB

CB was identified based on participants' self-reported medical history collected through a standardized questionnaire. During the survey, participants were asked, “Have you ever been told by a doctor or other healthcare professional that you have CB?” Those who responded “yes” were classified as having CB, ensuring that only cases diagnosed by qualified healthcare providers were included (25).

### 2.3 Measurement of HbEtO hemoglobin adducts

HbEtO is a reliable marker for cumulative EtO exposure due to its extended biological half-life of up to 4 months. This prolonged half-life allows HbEtO levels to reflect sustained exposure, making it suitable for assessing chronic exposure risks. In this study, HbEtO levels were measured to estimate participants' internal EtO dose. EtO hemoglobin adducts in red blood cells were quantified using a modified Edman reaction, following the protocols in the NHANES Laboratory Procedures Manual. Samples were preserved at −30°C to maintain accuracy, with any data irregularities reviewed rigorously by contract laboratories. Whole-blood hemoglobin content was measured through liquid chromatography-tandem mass spectrometry, while HbEtO was quantified using high-performance liquid chromatography (HPLC), with results reported in pmol/g Hb. This approach provides a dependable measure of EtO exposure through HbEtO levels (26, 27).

### 2.4 Covariates

Data were gathered through questionnaires, laboratory tests, and physical examinations, with covariate selection validated using a Directed Acyclic Graph (DAG) in Supplementary Figure 1 to ensure appropriate inclusion and minimize confounding. Key covariates included sex (female/male), age, and race (Mexican American, Other Hispanic, Non-Hispanic Black, Other Race). Education level was classified as less than high school, high school or equivalent, and college or higher. Poverty-to-income ratios (PIRs) were grouped into < 1.3, 1.3–3.5, and >3.5. Smoking status was defined as a lifetime consumption of over 100 cigarettes, and body mass index (BMI) was categorized into < 25, 25–30, and >30 kg/m^2^. Sedentary activity was classified based on self-reported duration as < 3 h, 3-6 h, or >6 h per day. Alcohol consumption was defined as four or more drinks within a 2-h period per day. Self-reported diagnoses of diabetes and hypertension were also included in the analysis (28–30).

### 2.5 Statistical analysis

NHANES sampling weights were applied following CDC guidelines to ensure accurate statistical analysis. Baseline characteristics were presented as means ± standard deviations for continuous variables, analyzed using weighted *t*-tests, and as proportions for categorical variables, assessed using chi-square tests. Missing data were managed through multiple imputation to enhance the robustness of the analysis. To examine the association between HbEtO levels and CB, we used logistic regression models with weighted variables. HbEtO levels were categorized into quartiles for trend analysis. Model 1 included no covariates, Model 2 adjusted for demographic variables, and Model 3 further controlled for alcohol consumption, education level, smoking status, BMI, PIR, diabetes, hypertension, and sedentary time. Due to the skewness in HbEtO data, we applied a logarithmic transformation (Log_2_HbEtO) to approximate a normal distribution (31). A weighted smoothing curve was fitted to assess any nonlinear relationship between Log_2_HbEtO and CB, with a threshold effect analysis to identify potential inflection points. Additionally, a subgroup analysis was conducted to explore potential effect modifiers in the Log_2_HbEtO-CB association (32). All statistical analyses were performed using R version 4.2.0 and Empower software (X&Y Solutions). A *p* < 0.05 was considered statistically significant (33).

## 3 Results

### 3.1 Baseline characteristics

Table 1 summarizes the baseline characteristics of the 5,044 participants. The sample is nearly evenly split by sex, with 50.2% female and 49.8% male, and has an average age of 48.2 years (±16.9). Non-Hispanic whites make up the majority (64.3%), and 6.1% of participants (307 individuals) reported CB. Key demographic differences are observed between those with and without CB: participants with CB are generally older, averaging 55.2 years compared to 47.7 years in those without CB, and include a higher proportion of females (64.4%) and Non-Hispanic whites (75.5%).

**Table 1:** Participants with/without chronic bronchitis.

**Characteristics**	**Tota**	**Without CB**	**With CB**	***P*-value**
	***N* = 5,044**	***N* = 4,737**	***N* = 307**	
Age, years	48.2 ± 16.9	47.7 ± 16.9	55.2 ± 16.0	< 0.001
Gender, %				< 0.001
Female	2,532 (50.2%)	2,331 (49.2%)	198 (64.4%)	
Male	2,512 (49.8%)	2,406 (50.8%)	109 (35.6%)	
Race/ethnicity, %				< 0.001
Mexican American	474 (9.4%)	464 (9.8%)	11 (3.5%)	
Other Hispanic	318 (6.3%)	303 (6.4%)	13 (4.1%)	
Non-Hispanic white	3,243 (64.3%)	3,017 (63.7%)	232 (75.5%)	
Non-Hispanic black	540 (10.7%)	512 (10.8%)	29 (9.3%)	
Other race	469 (9.3%)	445 (9.4%)	24 (7.8%)	
Education level, %				< 0.001
Under high school	691 (13.7%)	649 (13.7%)	42 (13.6%)	
High school or equivalent	1,211 (24.0%)	1,104 (23.3%)	104 (33.8%)	
College graduate or above	3,142 (62.3%)	2,984 (63.0%)	162 (52.7%)	
PIRs, %				< 0.001
< 1.3	1,004 (19.9%)	914 (19.3%)	87 (28.2%)	
1.3–3.5	2,073 (41.1%)	1,933 (40.8%)	142 (46.4%)	
>3.5	1,967 (39.0%)	1,890 (39.9%)	78 (25.4%)	
BMI (kg/m^2^), %				< 0.001
< 25	1,352 (26.8%)	1,307 (27.6%)	42 (13.8%)	
25–30	1,659 (32.9%)	1,549 (32.7%)	108 (35.3%)	
> 30	2,033 (40.3%)	1,881 (39.7%)	156 (50.9%)	
Smoking status, %				< 0.001
Yes	2,214 (43.9%)	1,999 (42.2%)	213 (69.4%)	
No	2,830 (56.1%)	2,738 (57.8%)	94 (30.6%)	
Alcohol consumption, %				0.001
< 4 drinks/day	4,333 (85.9%)	4,088 (86.3%)	245 (79.7%)	
≥4 drinks/day	711 (14.1%)	649 (13.7%)	62 (20.3%)	
Diabetes, %				< 0.001
Yes	540 (10.7%)	478 (10.1%)	59 (19.3%)	
No	4,504 (89.3%)	4,259 (89.9%)	248 (80.7%)	
Hypertension, %				< 0.001
Yes	1,654 (32.8%)	1,516 (32.0%)	142 (46.2%)	
No	3,390 (67.2%)	3,221 (68.0%)	165 (53.8%)	
Sedentary time, h, %				0.163
< 3 h	994 (19.7%)	947 (20.0%)	50 (16.2%)	
3–6 h	1,745 (34.6%)	1,630 (34.4%)	118 (38.5%)	
>6 h	2,305 (45.7%)	2,165 (45.7%)	139 (45.2%)	
Log_2_HbEtO, pmol/g Hb	4.8 ± 1.5	4.8 ± 1.5	5.3 ± 1.8	< 0.001

PIRs, poverty-to-income ratios; BMI, body mass index; Log_2_HbEtO, Natural Log_2_-transformed hemoglobin adducts of ethylene oxide; CB, chronic bronchitis.

Socioeconomic and health factors also differ between groups. Participants with CB tend to have lower education levels and lower PIRs, with 28.2% having a ratio below 1.3. They report higher rates of smoking (69.4%) and more frequent alcohol consumption, with 20.3% consuming four or more drinks per day. Health indicators reveal increased rates of diabetes (19.3%) and hypertension (46.2%) in the CB group, along with a higher prevalence of obesity (50.9% with a BMI above 30 kg/m^2^). Additionally, Log_2_HbEtO levels are significantly higher in the CB group (5.3 ± 1.8 pmol/g Hb) than in those without CB (4.8 ± 1.5 pmol/g Hb), suggesting a potential link between ethylene oxide exposure and CB risk.

### 3.2 The association between Log_2_HbEtO and CB

Table 2 demonstrates a strong association between Log_2_HbEtO levels and CB risk. In both Models 1 and 2, elevated Log_2_HbEtO levels were consistently linked to an increased risk of CB (Model 1: OR = 1.329, 95% CI: 1.243–1.420; Model 2: OR = 1.375, 95% CI: 1.282–1.474). After adjusting for all covariates in Model 3, each unit increase in Log_2_HbEtO was associated with a 24.3% greater likelihood of CB (OR = 1.243, 95% CI: 1.143–1.352). This association remained significant when Log_2_HbEtO levels were analyzed in quartiles. Across all models, participants in the highest quartile (Q4) had a markedly higher risk of CB than those in the lowest quartile (Q1) (*P* < 0.001). In Model 3, individuals in Q4 had more than twice the odds of CB compared to those in Q1 (OR = 2.096; 95% CI = 1.446–3.039, *P* < 0.001).

**Table 2:** Correlation between Log_2_HbEtO levels and the presence of CB.

	**Model 1**		**Model 2**		**Model 3**	
**Variable**	**(OR, 95% CI)**	***P*-value**	**(OR, 95% CI)**	***P*-value**	**(OR, 95% CI)**	***P*-value**
Log_2_HbEtO (continuous)	1.329 (1.243, 1.420)	< 0.001	1.375 (1.282, 1.474)	< 0.001	1.243 (1.143, 1.352)	< 0.001
Log_2_HbEtO (quartile)						
Q1 (< 3.99)	Reference		Reference		Reference	
Q2 (3.99-4.45)	0.971 (0.669, 1.409)	0.878	1.037 (0.711, 1.512)	0.851	1.035 (0.704, 1.522)	0.860
Q3 (4.45-5.44)	0.905 (0.621, 1.321)	0.606	1.064 (0.724, 1.565)	0.751	1.045 (0.705, 1.549)	0.828
Q4 (>5.44)	2.483 (1.811, 3.405)	< 0.001	3.125 (2.250, 4.339)	< 0.001	2.096 (1.446, 3.039)	< 0.001
*P* for trend	< 0.001		< 0.001		< 0.001	

Model 1: No covariates were adjusted. Model 2: adjusted for age, sex, race. Model 3: adjusted for age, sex, race, education level, PIRs, BMI, smoking status, alcohol consumption, hypertension, diabetes, and sedentary time.

Log_2_HbEtO, Natural Log_2_-transformed hemoglobin adducts of ethylene oxide.

### 3.3 Association between Log_2_HbEtO and CB in subgroups

Subgroup analyses explored the association between Log_2_HbEtO levels and CB across various demographic and socioeconomic groups, as shown in Table 3. The positive association between elevated Log_2_HbEtO levels and CB was statistically significant in most subgroups. However, certain groups, including individuals with a poverty-to-income ratio (PIR) above 3.5, Mexican Americans, Non-Hispanic Blacks, Other Hispanics, and those from other racial backgrounds, did not show significant associations (*P* > 0.05). These findings suggest that socioeconomic and racial factors may influence the relationship between Log_2_HbEtO and CB, potentially due to varying levels or sources of exposure. Moreover, none of the covariates significantly modified the Log_2_HbEtO-CB relationship, as indicated by interaction terms with *P*-values exceeding 0.05 for all variables. This lack of significant interaction suggests that while there are overall trends, no specific covariate substantially alters the effect of Log_2_HbEtO on CB across these subgroups.

**Table 3:** Subgroup analysis of the correlation between Log_2_HbEtO levels and CB.

**Characteristics**	**CB [β (95%CI)]**	***P*-value**	***P* for interaction**
Age			0.568
≤ 60 years	1.20 (1.08, 1.33)	< 0.001	
>60 years	1.25 (1.11, 1.41)	< 0.001	
Gender			0.262
Female	1.26 (1.14, 1.40)	< 0.001	
Male	1.17 (1.04, 1.31)	0.008	
Race/ethnicity			0.274
Mexican American	0.79 (0.52, 1.21)	0.280	
Other Hispanic	1.15 (0.79, 1.68)	0.459	
Non-Hispanic White	1.26 (1.13, 1.40)	< 0.001	
Non-Hispanic Black	1.22 (1.00, 1.49)	0.050	
Other race	1.32 (0.98, 1.79)	0.069	
PIRs			0.135
< 1.3	1.31 (1.14, 1.49)	< 0.001	
1.3–3.5	1.23 (1.10, 1.39)	< 0.001	
>3.5	1.01 (0.80, 1.26)	0.955	
BMI (kg/m^2^)			0.503
< 25	1.25 (1.05, 1.48)	0.013	
25–30	1.29 (1.12, 1.49)	< 0.001	
> 30	1.16 (1.04, 1.30)	0.010	
Smoking status			0.400
Yes	1.20 (1.10, 1.30)	< 0.001	
No	1.33 (1.05, 1.69)	0.017	
Alcohol consumption			0.778
< 4 drinks/day	1.21 (1.10, 1.33)	< 0.001	
≥4 drinks/day	1.24 (1.07, 1.45)	0.006	
Diabetes			0.313
Yes	1.33 (1.12, 1.57)	0.001	
No	1.20 (1.09, 1.32)	< 0.001	
Hypertension			0.364
Yes	1.26 (1.13, 1.41)	< 0.001	
No	1.17 (1.05, 1.31)	0.006	
Sedentary time, h			0.838
< 3 h	1.25 (1.03, 1.52)	0.026	
3–6 h	1.18 (1.04, 1.35)	0.013	
>6 h	1.24 (1.11, 1.40)	< 0.001	

PIRs, poverty-to-income ratios; Log_2_HbEtO, Natural Log_2_-transformed hemoglobin adducts of ethylene oxide; BMI, body mass index; CB, chronic bronchitis.

### 3.4 Sensitivity analysis of key determinants in CB risk

Using logistic regression with robust standard errors, the analysis in Supplementary Table 2 reveals a significant association between Log_2_HbEtO levels and the risk of CB. Higher Log_2_HbEtO levels were linked to an increased likelihood of CB (coefficient = 0.202, *P* < 0.001). This method was chosen to improve the precision of the findings by controlling for data variability and minimizing the influence of outliers.

This association was further influenced by individual characteristics. Males and older adults showed a higher risk of CB (gender coefficient = 0.601, *P* = 0.001; age coefficient = 0.026, *P* < 0.001). Additionally, body mass index (BMI) and economic status, represented by the Poverty Index Ratio (PIR), were significant factors. Higher BMI was positively associated with CB risk (BMI coefficient = 0.049, *P* < 0.001), while a higher PIR, reflecting better economic status, was associated with a reduced likelihood of CB (PIR coefficient = −0.204, *P* = 0.002). Smoking status was also an important factor, with findings underscoring its role in elevating CB risk (smoking coefficient = −0.517, *P* = 0.014).

### 3.5 Nonlinear relationship between Log_2_HbEtO and CB

**Figure 2:**
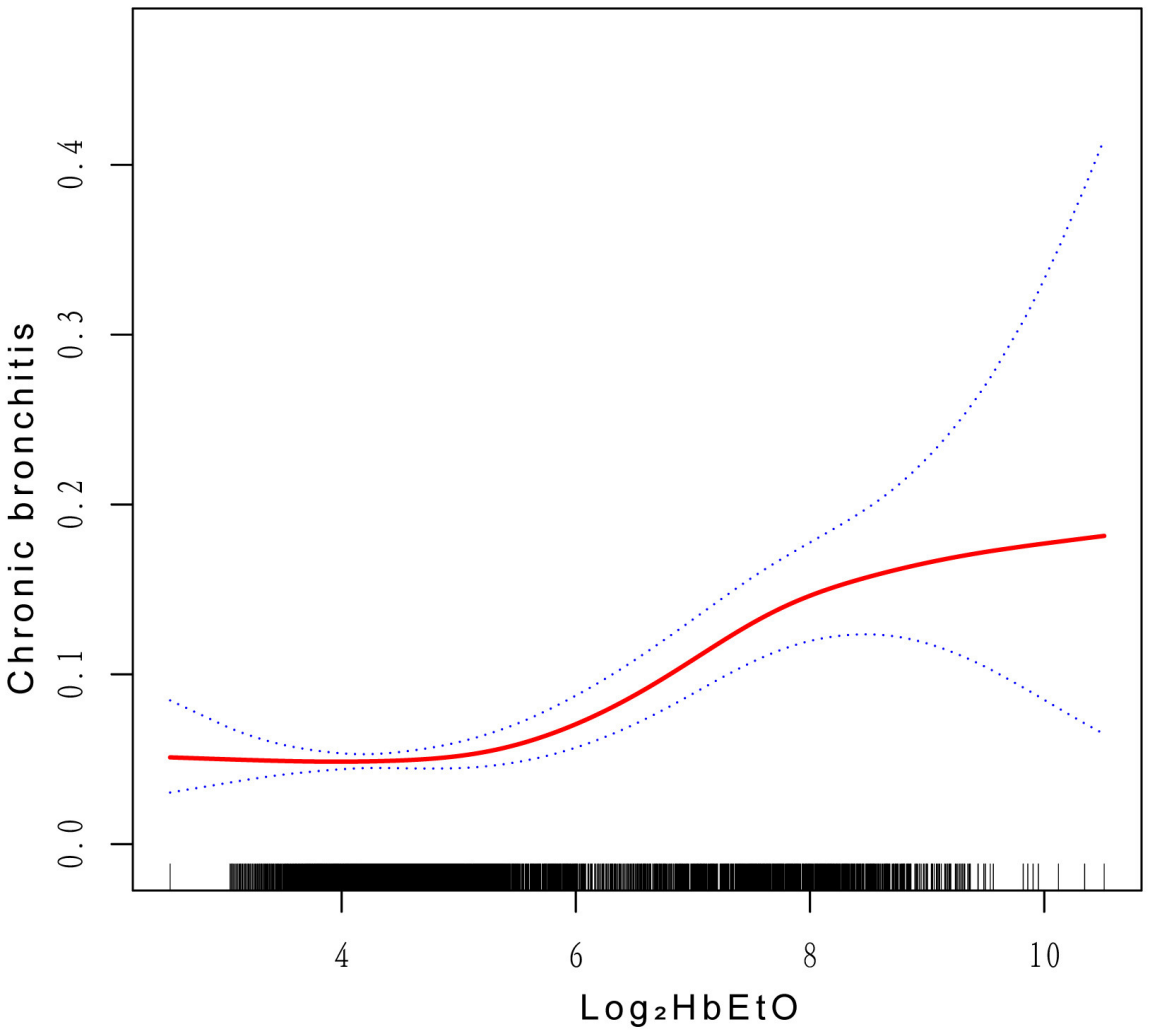
Correlations between Log_2_HbEtO levels and CB. The y-axis represents the proportion or risk of CB, with values ranging from 0 to 1. The x-axis represents Natural Log_2_-transformed hemoglobin adducts of ethylene oxide levels.

The fully adjusted model reveals a smooth curve indicating a significant positive association between Log_2_HbEtO levels and CB risk, displaying a U-shaped nonlinear pattern (*P* < 0.001) (Figure 2). This pattern suggests that CB risk increases at both lower and higher levels of Log_2_HbEtO, underscoring the complexity of this relationship. A critical inflection point was identified at a Log_2_HbEtO level of 4.87; beyond this threshold, CB risk rises markedly. These findings highlight the importance of monitoring and managing EtO exposure to effectively reduce the risk of CB.

## 4 Discussion

This cross-sectional survey is the first to examine the relationship between CB prevalence and Log_2_HbEtO levels. After accounting for all covariates, a positive association between CB and Log_2_HbEtO was identified. The fitted smoothed curves reveal a U-shaped pattern, indicating that both low and high levels of Log_2_HbEtO are linked to increased CB risk. Additionally, the analysis of saturation and threshold effects identified an inflection point at a Log_2_HbEtO level of 4.87. These findings underscore the potential impact of Log_2_HbEtO on CB and highlight the importance of monitoring and mitigating its adverse effects on health.

Previous studies have highlighted the serious health effects of ethylene oxide (EtO) exposure (34). The International Agency for Research on Cancer (IARC) has classified EtO as a Group 1 carcinogen based on data from epidemiological and animal studies (35). Numerous investigations confirm a strong association between EtO exposure and the risk of developing various cancers (34–37). In animal studies, EtO exposure has been linked to significantly lower antioxidant levels, which can lead to the development of malignant tumors in multiple organs (38). Additionally, several cross-sectional studies have connected EtO exposure to health issues such as obesity, diabetes, depression, and hypertension (26, 31, 39, 40). Research has also shown that EtO exposure increases the risk of chronic respiratory diseases (28). In the general U.S. population, greater EtO exposure correlates with a higher risk of asthma (27). Studies, such as those by Klonne et al., have reported that long-term inhalation of EtO causes lung fibrosis in F-344 rats, while findings from the U.S. (41). National Toxicology Program indicate a significant association between lung cancer prevalence in B6C3F1 mice and prolonged EtO exposure (42), also noting its link to inflammation (38, 43). Our study also identified an association between EtO exposure and CB, supported by a U-shaped pattern in the smoothed curve, which suggests that EtO exposure increases the risk of CB. The strength of this relationship is further underscored by consistent results in subgroup analyses. However, the lack of statistically significant associations in certain subgroups, such as those with higher poverty-to-income ratios or specific racial and ethnic backgrounds, may indicate differences in exposure sources or levels. For instance, individuals with higher incomes or from certain racial/ethnic groups may experience unique exposure characteristics or occupational risks that influence their susceptibility to EtO. Furthermore, smaller sample sizes within some subgroups could have limited statistical power, leading to non-significant *P*-values even when trends suggest a potential association. These findings emphasize the need to consider population diversity when evaluating the health effects of EtO exposure. Future studies should further explore these subgroup differences to better understand the implications of EtO on CB risk.

The findings of this study highlight the need for further research to fully understand the relationship between ethylene oxide (EtO) exposure and CB. A consistent positive association was observed, indicating that the prevalence of CB may significantly rise with increasing levels of EtO exposure. Notably, a strong correlation between EtO exposure and tobacco exposure was found, suggesting that direct contact with tobacco and its emitted EtO heightens the risk of bronchial diseases (44). The specific mechanisms behind this association warrant additional investigation. EtO exposure may lead to oxidative stress, which depletes cytoplasmic content and results in cell shrinkage, ultimately damaging vital organs and triggering bronchial inflammation. The detrimental effects of this oxidative stress can significantly impact health. Furthermore, research by Lynch et al. demonstrated that EtO exposure causes inflammation in the locomotor organs of rodents, suggesting that the inflammatory response associated with EtO exposure may negatively affect bronchial health (39, 43, 45). However, the precise mechanisms at play still require further exploration.

This study utilized data from approximately 29,400 NHANES participants collected between 2013 and 2018, ultimately analyzing data from 5,044 individuals after excluding those with missing information on HbEtO and CB. As detailed in Supplementary Table 1, individuals lacking HbEtO or CB data were significantly younger than those included in the final analysis, with notable differences in age, sex, and race. The observed age differences among those with missing data may stem from several factors. First, younger individuals are generally less exposed to occupational or environmental HbEtO, which may lead to a lower priority for data collection in this demographic. Second, NHANES data collection relies heavily on self-reported information and follow-up, and health assessments for younger participants may be deprioritized due to the assumption that their direct health risks are lower. Third, NHANES typically targets specific exposure tests at individuals with known risk factors, resulting in higher rates of missing data among those considered lower risk, such as younger individuals.

This study utilized data from a U.S. population sample to examine the association between ethylene oxide (EtO) exposure and CB, offering valuable insights into the respiratory impact of this widely used industrial compound. To enhance the reliability of our findings, we conducted subgroup analyses and carefully adjusted for key confounders. Nonetheless, several limitations warrant consideration. First, the reliance on self-reported CB data may introduce recall bias and potential misclassification. Additionally, the cross-sectional study design limits causal interpretation, as it does not allow for establishing the temporal sequence between EtO exposure and CB onset. Furthermore, the NHANES database lacks specific information on respiratory infections and occupational history, limiting our ability to assess infection-related risks or distinguish between occupational and environmental EtO exposure sources. Although adjustments were made for smoking status, residual confounding may still exist due to unmeasured factors such as smoking intensity, duration, and passive exposure. Also, since EtO levels may vary over time, a single measurement may not adequately reflect cumulative exposure. Future research, particularly longitudinal studies, is essential to deepen our understanding of EtO exposure's impact on CB risk and to address these limitations.

## 5 Conclusion

The findings of this study indicate a significant association between EtO exposure and CB, with a fitted smoothing curve revealing a U-shaped association pattern. However, the cross-sectional design limits causal inference, and unmeasured confounders, such as exposure to other environmental pollutants, may have influenced the results. Additionally, HbEtO, while used as a marker for EtO exposure, may not fully capture long-term exposure, potentially leading to misclassification. Although the NHANES sample is not globally representative, this study underscores the importance of monitoring and controlling EtO exposure to reduce its potential adverse effects on respiratory health. Future research should investigate the specific mechanisms through which EtO exposure impacts bronchial health and seek to validate and expand upon these findings.

## Data Availability

Publicly available datasets were analyzed in this study. This data can be found here: https://wwwn.cdc.gov/nchs/nhanes.
