# Supplementary material for: Associations between ethylene oxide exposure and chronic bronchitis: results from the NHANES 2013–2018
Source: Front Public Health. 2024 Dec 9;12:1424555. doi: 10.3389/fpubh.2024.1424555 (PMC11663886; doi:10.3389/fpubh.2024.1424555)

**Supplementary Material**

**Detailed Description of NHANES Sampling Methodology (NHANES 2013–2018)**

**1. Overview of NHANES Study Design**

The National Health and Nutrition Examination Survey (NHANES) is a comprehensive study led by the Centers for Disease Control and Prevention (CDC) that assesses the health and nutritional status of the U.S. population. NHANES uses a stratified multistage probability sampling technique to ensure that the sample is nationally representative of the U.S. population. This technique captures individuals from a wide range of geographic, socioeconomic, and racial/ethnic backgrounds.

**2. Stratified Sampling**

NHANES stratified sampling first divides the population into subgroups (strata) based on key variables, such as age, gender, and race/ethnicity. The population is stratified to ensure that smaller groups, such as racial minorities, are adequately represented in the sample. U.S. Census data are used to guide the stratification process. Oversampling of smaller subpopulations ensures sufficient sample sizes for robust statistical analysis.

**3. Multistage Sampling**

NHANES employs a multistage sampling approach. In the first stage, primary sampling units (PSUs) are selected, which are counties or groups of counties across the U.S. In the second stage, specific blocks or segments within these PSUs are randomly selected. In the third stage, households within these blocks are randomly chosen, and finally, individuals are selected from within households for participation.

**4. Sampling Weights and Representativeness**

NHANES assigns sampling weights to adjust for unequal probabilities of selection and to account for oversampling of certain subpopulations. These weights are also adjusted to correct for nonresponse bias. By applying these weights, the results are more representative of the entire U.S. population, taking into account age, gender, race, and socioeconomic factors.

**5. Data Collection Procedures**

NHANES data are collected through a combination of interviews, physical examinations, and laboratory tests. This rigorous approach enables comprehensive data collection on a wide array of health indicators. The combination of objective health measures and detailed survey data allows for robust analysis of associations between health outcomes and environmental or lifestyle exposures, such as ethylene oxide.

**References**

Centers for Disease Control and Prevention (CDC). NHANES 2013–2018 Reports. Available from: <https://wwwn.cdc.gov/nchs/nhanes/>.

**Supplement table 1** Results of odds ratio (OR) and 95% confidence interval analysis of the difference between Log_2_HbEtO levels and CB missing data groups and the non-missing data group.

| **Character** | Mean (No Missing) | Mean (Missing) | 95% CI | *P*-Value |
| --- | --- | --- | --- | --- |
| Age (years) | 50.07 | 28.85 | 20.65, 21.79 | <0.001 |
| Gender | 1.51 | 1.51 | -0.02, 0.01 | 0.854 |
| Race/ethnicity | 3.13 | 3.10 | -0.01, 0.07 | 0.161 |
| Education level | 3.49 | 3.50 | -0.05, 0.03 | 0.740 |

**Supplement table 2** Association of Log_2_HbEtO levels and key covariates with CB risk: logistic regression with robust standard errors.

Abbreviations: Coef., coefficient; Std. Err., standard Error; Log_2_HbEtO, natural Log-transformed hemoglobin adducts of ethylene oxide; PIRs, poverty-to-income ratios; BMI, body mass index; CB, Chronic Bronchitis;

| **Variable** | **Coef.** | **Std. Err.** | **Z-value (z)** | ***P*-value** | **95% CI Lower** | **95% CI Upper** |
| --- | --- | --- | --- | --- | --- | --- |
| const | -7.166 | 1.045 | -6.857 | <0.001 | -9.214 | -5.117 |
| Log_2_HbEtO | 0.202 | 0.057 | 3.562 | <0.001 | 0.091 | 0.314 |
| Gender | 0.601 | 0.176 | 3.423 | 0.001 | 0.257 | 0.945 |
| Age (years) | 0.026 | 0.007 | 3.968 | <0.001 | 0.013 | 0.039 |
| Race/ethnicity | 0.137 | 0.065 | 2.107 | 0.035 | 0.010 | 0.265 |
| Education level | 0.035 | 0.091 | 0.386 | 0.700 | -0.143 | 0.213 |
| BMI (kg/m²) | 0.049 | 0.010 | 5.152 | <0.001 | 0.031 | 0.068 |
| PIRs | -0.204 | 0.067 | -3.062 | 0.002 | -0.334 | -0.073 |
| Alcohol consumption | -0.001 | 0.001 | -0.628 | 0.530 | -0.004 | 0.002 |
| Hypertension | 0.006 | 0.189 | 0.033 | 0.974 | -0.365 | 0.377 |
| Diabetes | 0.062 | 0.180 | 0.347 | 0.729 | -0.291 | 0.416 |
| Smoking status | -0.517 | 0.210 | -2.464 | 0.014 | -0.928 | -0.106 |

**Supplement figure 1** Directed Acyclic Graph (DAG) Depicting the Relationship Between Log_2_HbEtO and Chronic Bronchitis.


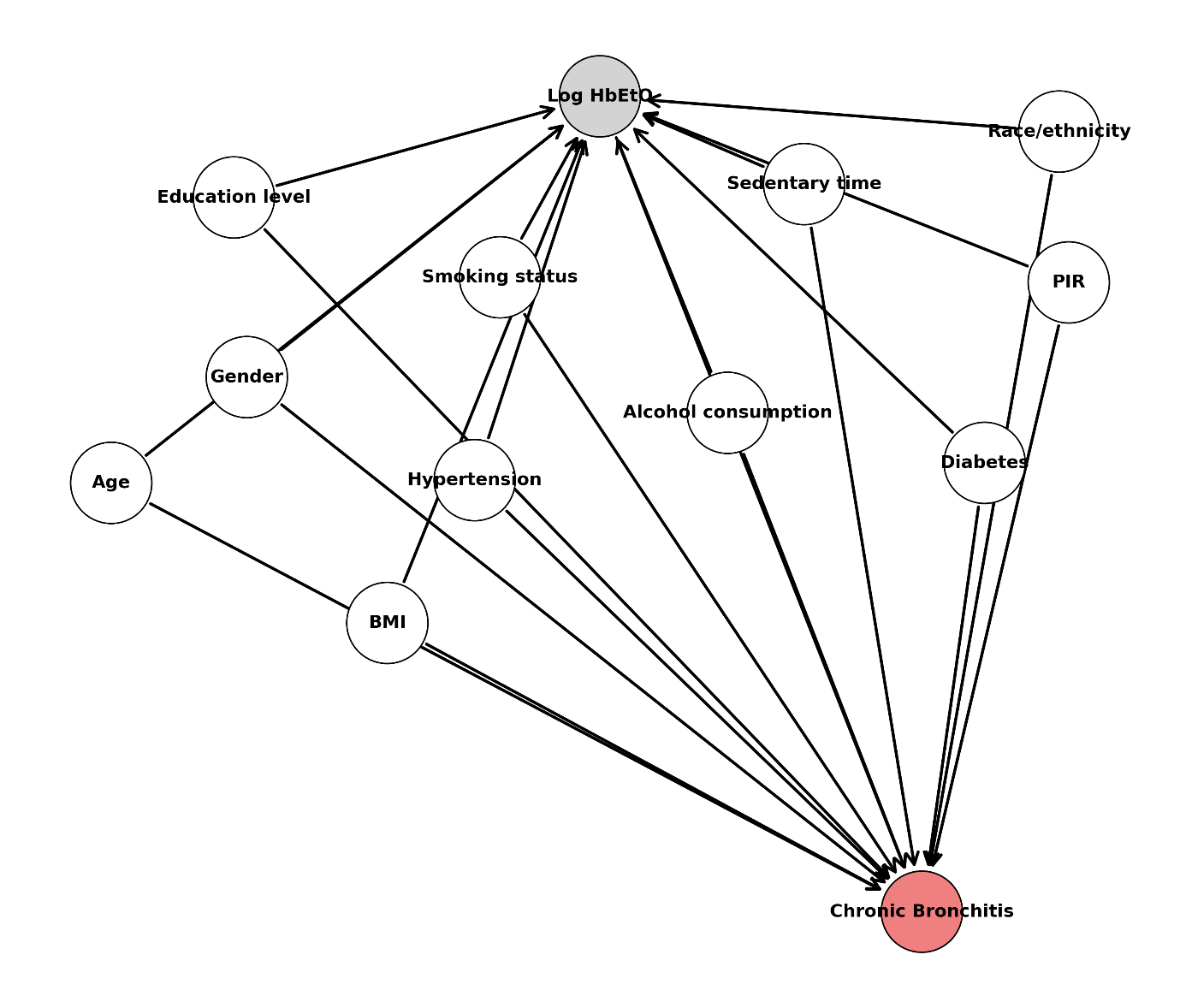

Supplement: Supplementary file 1 [file Data_Sheet_1.docx]
